# Biobased Acrylic Latexes/Sodium Carboxymethyl Cellulose Aqueous Binders for Lithium-Ion NMC 811 Cathodes

**DOI:** 10.1021/acsapm.3c02167

**Published:** 2024-01-08

**Authors:** Ana Clara Rolandi, Aitor Barquero, Cristina Pozo-Gonzalo, Iratxe de Meatza, Nerea Casado, Maria Forsyth, Jose R. Leiza, David Mecerreyes

**Affiliations:** † Institute for Frontier Materials, Deakin University, Melbourne, Victoria 3125, Australia; ‡ CIDETEC Basque Research and Technology Alliance (BRTA), Paseo Miramon 196,Donostia-San Sebastian 20014, Spain; § POLYMAT and Applied Chemistry Department, Faculty of Chemistry, University of the Basque Country UPV/EHU, Joxe Mari Korta center, Donostia-San Sebastián 20018, Spain; ∥ IKERBASQUE, Basque Foundation for Science, Bilbao 48009, Spain

**Keywords:** biobased latexes, renewable resources, waterborne binder, aqueous processing, NMC 811 cathodes, sustainability, lithium-ion batteries

## Abstract

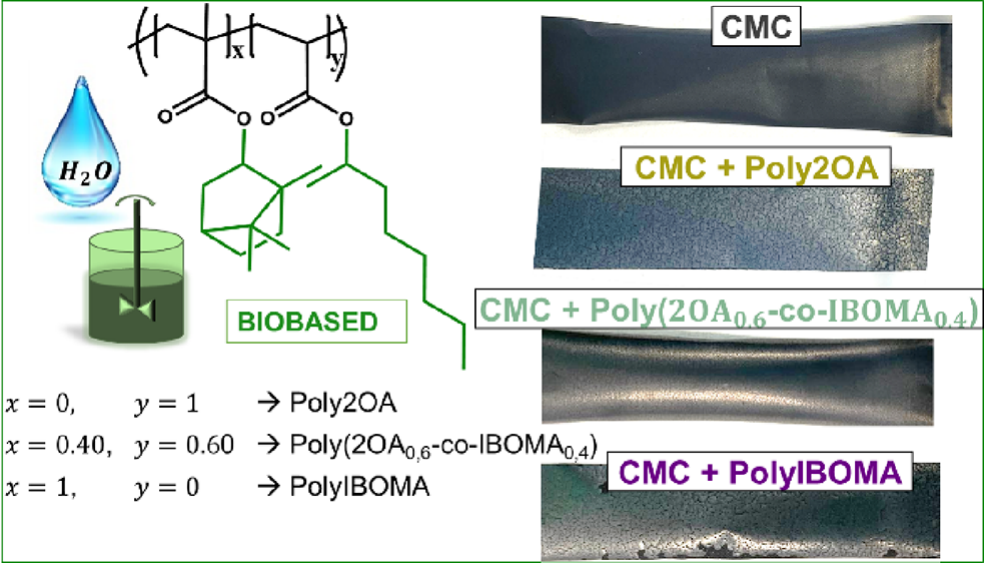

The increasing demands for sustainable energy storage technologies have prompted extensive research in the development of eco-friendly materials for lithium-ion batteries (LIBs). This research article presents the design of biobased latexes, which are fluorine-free and rely on renewable resources, based on isobornyl methacrylate (IBOMA) and 2-octyl acrylate (2OA) to be used as binders in batteries. Three different compositions of latexes were investigated, varying the ratio of IBOMA and 2OA: (1) Poly2OA homopolymer, (2) Poly(2OA_0,6_-*co*-IBOMA_0,4_) random copolymer, and (3) PolyIBOMA homopolymer. The combination of the two monomers provided a balance between rigidity from the hard monomer (IBOMA) and flexibility from the soft one (2OA). The study evaluated the performance of the biobased latexes using sodium carboxymethyl cellulose (CMC) as a thickener and cobinder by fabricating LiNi_0.8_Mn_0.1_Co_0.1_O_2_ (NMC 811) cathodes. Also, to compare with the state of the art, organic processed PVDF electrodes were prepared. Among aqueous slurries, rheological analysis showed that the CMC + Poly(2OA_0,6_-*co*-IBOMA_0,4_) binder system resulted in the most stable and well-dispersed slurries. Also, the electrodes prepared with this latex demonstrated enhanced adhesion (210 ± 9 N m^–1^) and reduced cracks compared to other aqueous compositions. Electrochemical characterization revealed that the aqueous processed cathodes using the CMC + Poly(2OA_0,6_-*co*-IBOMA_0,4_) biobased latex displayed higher specific capacities than the control with no latex at high C-rates (100.3 ± 2.1 vs 64.5 ± 0.8 mAh g^–1^ at 5C) and increased capacity retention after 90 cycles at 0.5C (84% vs 81% for CMC with no latex). Overall, the findings of this study suggest that biobased latexes, specifically the CMC + Poly(2OA_0,6_-*co*-IBOMA_0,4_) composition, are promising as environmentally friendly binders for NMC 811 cathodes, contributing to the broader goal of achieving sustainable energy storage systems.

## Introduction

Nowadays, lithium-ion batteries (LIBs) are one of the most appealing technologies for the energy storage system of electric vehicles (EVs), given their high efficiency, long cycle life, and high power density. Polyvinylidene fluoride (PVDF) is the most common binder used to process cathode electrodes of actual LIBs given its chemical and thermal stability.^1^ However, this fluoropolymer has several drawbacks, being difficult to dispose of and needing to be dissolved in *N*-methylpyrrolidone (NMP) during the electrode fabrication, which is a flammable and toxic solvent.^2^ Also, PVDF requires of high-processing temperature during electrode drying (110 °C against 60 °C for aqueous electrodes), which is disadvantageous in terms of energy consumption.^3^ Therefore, research efforts have been conducted toward the development of fluorine-free and also water-soluble binders to achieve environmentally friendly, low cost, and high-performance LIBs.

The most used water-soluble binder is the biopolymer sodium carboxymethyl cellulose (CMC), due to its dispersion ability and strong interaction with the active and conductive material.^4^ However, the CMC lacks flexibility and has poor adhesion to the current collector, resulting in brittle electrodes with cracks and delamination. To solve this issue, in the anodes, CMC is normally blended with styrene butadiene rubber (SBR),^5^ which thanks to its elastomeric properties provides high adhesion to the current collector and cohesion of the anode active layer for optimum electrode manufacturing and cycling lifetime under battery operating conditions.^6,7^ Unfortunately, this is not the same scenario for high-voltage cathodes, since SBR latex oxidizes at high potentials limiting the battery cycling conditions.^8^ Therefore, other types of commercial latexes have been used in combination with CMC for the aqueous processing of cathodes such as a fluorine acrylic copolymer latex (TRD 202A)^9−12^ and waterborne polyvinylidene fluoride (PVDF)^13^ or polytetrafluoroethylene (PTFE) latexes.^14−16^ Although these latexes avoid the use of NMP and lessen the environmental effect, the presence of fluoride compounds also hinders the recycling and disposal of the battery once used.^17^ The extraction of fluorinated binders from the electrodes requires thermal treatment, which releases highly toxic fluorocarbons (such as hexafluoropropene, fluorophosgene, cycloperfluorobutane, perfluoroisobutene, etc.) that contribute to ozone layer depletion.^18^ In this regard, fluorine-free latexes have been explored as binders for cathodes, such as polyacrylic latexes,^15,19,20^ amphiphilic cross-linked binders based on poly(ethylene glycol), *n*-butyl acrylate, and acetone acrylamide^21^ or polyvinyl acetate.^22,23^ However, most of these fluorine-free latexes still rely on fossil fuel resources.

As a result of growing environmental concerns, the use of raw materials derived from biomass or other renewable resources has become increasingly important as a sustainable replacement for traditional petroleum-based or fluorine-containing binders by biobased ones. Herein, we present (meth)acrylic latexes obtained by the copolymerization of isobornyl methacrylate (IBOMA) and 2-octyl acrylate (2OA). Both monomers are commercially available with a biocontent of 71 and 73%.^24,25^ The homopolymers of Poly2OA and PolyIBOMA present glass-transition temperatures of −44^26^ and 140–195 °C,^25^ respectively. Consequently, the copolymerization of them in the right proportions can yield a copolymer with intermediate properties, such as good adhesion while maintaining internal cohesion. In this work, three different latexes were studied as binders for the LiNi_0.8_Mn_0.1_Co_0.1_O_2_ (NMC 811) cathode based on Poly2OA and PolyIBOMA homopolymers and one random copolymer (Poly(2OA_0,6_-*co*-IBOMA_0,4_)). In all cases, the biobased latex was combined with CMC as a thickener and cobinder in the water processing of NMC 811 cathodes. As control with no latex in the composition, other slurries using CMC and PVDF were prepared, using water and NMP as a solvent, respectively.

## Experimental Section

### Materials

For the synthesis of the latexes, styrene (S, Quimidroga), 2-octyl acrylate (2OA, Polykey), and potassium persulfate (KPS, Sigma-Aldrich) were used as purchased. Isobornyl methacrylate (IBOMA, Evonik) and Dowfax 2A1 (Dow Chemicals) were kindly supplied by their respective manufacturers and used as received. Deionized water was used in all reactions.

For the electrode fabrication, sodium carboxymethyl cellulose (CMC, 250,000 molecular weight, Sigma), poly(vinylidene fluoride) (PVDF, 534,000 molecular weight, Sigma-Aldrich), 1-methyl-2-pyrrolidone (NMP, ≥ 99%, Sigma-Aldrich), conductive carbon C-NERGY Super C45 (C45, Imerys), washed LiNi_0.8_Mn_0.1_Co_0.1_O_2_ (NMC 811, Targray), and carbon-coated aluminum current collector (CC-Al, Gelon) were used as received. Also, for the negative electrode preparation, graphite (Hitachi HE3) and styrene butadiene rubber (SBR, BM451B, Zeon) were used as received.

### Synthesis of Biobased Latexes

Three different latexes were synthesized using the biobased monomers. Poly2OA and Poly(2OA_0,6_-*co*-IBOMA_0,4_) were synthesized by seeded semibatch emulsion polymerization, while PolyIBOMA was synthesized by batch miniemulsion polymerization. In both cases, KPS was used as the thermal initiator and Dowfax 2A1 as the anionic surfactant.

The seeded semibatch emulsion polymerizations were carried out in a 250 mL jacketed glass reactor, equipped with a mechanical turbine stirrer, a N_2_ inlet, a condenser, and a sampling device. The reaction temperature (70 °C) was controlled with a thermostatic bath. The polymerizations were carried out in two steps. In the first step, a polystyrene (PS) latex was synthesized by batch emulsion polymerization to be used as a seed, and then, the seed was grown with the polymer of interest in a semibatch emulsion polymerization step. A feeding of a preemulsion (mixture of water, monomer, and surfactant) was fed during 3 h for Poly2OA and 4 h for Poly(2OA_0,6_-*co*-IBOMA_0,4_). In all cases, an additional hour was given to the reaction to reach full conversion. At the end of the polymerization, the seed polymer consisted on less than 5 wt % of the polymer.

The polymerization of PolyIBOMA was carried out by batch miniemulsion polymerization in a 100 mL bottle immersed in a water bath at 70 °C. The entire load was charged in the bottle, and then, it was sealed. Then, the bottle was purged with nitrogen for 10 min. After it was immersed in the bath, the bottle was tumbled end-overend at 49 rpm for 3 h. The bottle was left to cool to room temperature before it was opened.

### Biobased Latex Characterization

The thermal properties were characterized by differential scanning calorimetry (DSC). The experiments were performed using a PerkinElmer 8000 DSC instrument equipped with an Intracooler II and calibrated with indium and tin standards. The heating rate was 10 °C min^–1^ in the temperature range of −80 to 100 °C, and between 5 and 10 mg of dry sample was used every time. The measurements were performed by sealing the samples in aluminum pans. The samples were first heated from room temperature to 100 °C to erase thermal history and then cooled, and finally, a second heating was performed. Note that only *T*_g_ of the copolymer was measured.

Z-Average particle diameters were measured by dynamic light scattering (DLS). A Zetasizer Nano ZS instrument from Malvern Instruments was used. Samples were prepared by diluting a fraction of the latex in doubly deionized water. The analyses were carried out at 25 °C and a run consisted of 2 min of temperature equilibration followed by three size measurements of 2 min each. An average value is given as a result.

The soluble and nonsoluble (gel) fraction of the polymers was separated by Soxhlet extraction. A glass fiber pad (CEM) was weighted. A few drops of latex were put in the pad right after the samples were withdrawn and dried in an oven at 65 °C overnight. The glass fiber pad with the polymer was weighed again before performing a continuous extraction with THF under reflux in the Soxhlet for 24 h. The glass fiber pad was dried in an oven at 65 °C overnight and weighted. The gel content was calculated as the weight loss.

The soluble fractions were dissolved in GPC-grade THF at a concentration of about 3 mg/mL. Then, the solution was filtered (polyamide Φ = 45 μm) before being injected into the SEC via an autosampler (Waters 717, Milford, MA). A pump (LC-20A; Shimadzu, Japan) controlled a THF flow of 1 mL/min. The GPC was composed of a differential refractometer (Waters 2410, Milford, MA) and three columns in series (Styragel HR2, HR4, and HR6, with pore sizes ranging from 10^2^ to 10^6^ Å). Measurements were performed at 35 °C. Molecular weights were determined using a calibration curve based on polystyrene standards. The measured gel contents, weight-average molar mass, and dispersities are reported in Table S1.

### Electrode Preparation

Cyclic voltammetry (CV) was performed on the copolymer biobased latex without incorporating any active material. The primary objective was to identify any redox reactions or irreversible processes exhibited by the latex, potentially affecting the electrochemical behavior of the battery. To conduct this analysis, electrodes were exclusively prepared with the latex binder, CMC, and conductive carbon in a mass ratio of 50:25:25, respectively. Coin cells were assembled using these electrodes, with lithium foil as the anode and 100 μL of 1 M LiPF_6_ in EC:DMC (1:1) as the electrolyte. Following an 8 h stabilization period at an open-circuit potential, the coin cells were subjected to CV analysis. A VMP-3 potentiostat (Biologic Science Instruments) was utilized, scanning within the ranges of 3.0–4.5 and 3.0–5.0 V (vs Li/Li^+^) at 0.1 mV s^–1^. The potential was linearly varied with time, and the resulting current response was recorded.

Cathode slurries of 50 g of solids were composed of 90 wt % NMC 811, 5 wt % carbon black, 2 wt % CMC, and 3 wt % of biobased latexes. For the sake of comparison, two more slurries were prepared used as a control: first, one slurry replacing the 3 wt % of biobased latex by CMC (therefore, 5 wt % of CMC) and a second one using 5 wt % of PVDF as a binder. The latter slurry was prepared using NMP as the solvent instead of water. The CMC or PVDF was previously dissolved in the corresponding solvent until a concentration of 5 wt % was obtained. The biobased latex was then added in the corresponding slurry to ensure an optimal distribution. The solid content of the latexes was 45, 40, and 23 wt % for Poly2OA, Poly(2OA_0,6_-*co*-IBOMA_0,4_), and PolyIBOMA, respectively. Therefore, the amount of binder was adjusted to add the same latex quantity (1.5 g of solids) to the slurries. Water is added to standardize the final solid/liquid ratio to 1/0.90 for all slurries. Afterward, the conductive carbon and the active material were mixed for 4 h using mechanical mixing at a high rate (450–550 rpm) to obtain a homogeneous slurry. The slurry was then applied using a doctor blade (90 mm min^–1^) and metallic stainless steel applicators to a carbon-coated aluminum (CC-Al) current collector, controlling the thickness to achieve a loading of 2.0–2.1 mAh cm^–2^. Finally, the electrodes were dried in a convection oven at 60 °C for 1 h and then calendared using a roll press from DMP solutions until a density between 2.65 and 2.70 g cm^–3^ was reached, corresponding to a theoretical porosity of 40–42%. The theoretical porosity (ϕ) was determined by considering the density of the different materials: 4.94 g cm^–3^ for NMC 811, 1.89 g cm^–3^ for C45, 1.62 g cm^–3^ for PDADMA-DEP, 1.41 g cm^–3^ for PDADMA-DBP, 1.59 g cm^–3^ for CMC, and 1.74 g cm^–3^ for PVDF, along with their respective proportion in the total cathode formulation. Using this data, the density of an electrode with a 0% porosity (ρ_0%_) can be calculated as follows: ρ_0%_ = 0.9 × ρ_NMC811_ + 0.05 × ρ_*C*45_ + 0.05 × ρ_binder_. Conversely, the real density (ρ_real_) of the electrode can be calculated by measuring its mass and thickness. Finally, the theoretical porosity the electrode can be derived from the formula: . In this study, theoretical porosity was standardized to the range between 40 and 42%. From these parameters, the ideal thickness to which the electrode should be calender was determined, ensuring the targeted porosity was achieved. To obtain circular electrodes with a 16.6 mm diameter, the electrodes were last punched with a disk cutter.

The same process used for the cathodes was applied to the graphite anodes. The composition consisted of 94 wt % graphite, 2 wt % carbon black, 2 wt % CMC, and 2 wt % SBR latex. In order to obtain full cells with a negative-to-positive capacity ratio (N/P) of 1.1, the mass loading of the anodes was set to be 2.2–2.3 mAh cm^–2^. In this instance, the anodes were cut into circular electrodes with a diameter of 17.7 mm (diameter circular electrodes).

### Slurry and Electrode Characterization

Rheology measurements were performed on the slurry, prior to casting it on the current collector. Using a rheometer AR 200ex (TA Instruments), dynamic rheological studies between 0.1 and 1000 s^–1^ shear rate were made at 25 °C using a parallel plate geometry (40 mm diameter and 1 mm gap setting). The rheology results can be studied with the power-law eq 1:^27^
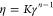
1where η is the viscosity, γ the shear rate, *K* is a consistency constant, and *n* is a factor that quantifies the similarity to a Newtonian fluid (where *n* = 1).

The microstructural characteristics of the different electrodes and dispersion of the components were observed by field emission scanning electron microscope (FESEM, ULTRA plus ZEISS), and no coatings were applied to the electrode surfaces. Finally, to gauge the adhesion of the coatings to the current collector in the presence of the biobased latex, peeling tests were performed. To achieve this, electrode strips of 2 × 9 cm were adhered on methacrylate plates and pulled in a 90° angle at 20 mm min^–1^, obtaining the adhesion strength value (N m^–1^). The 90° peel tests are carried out in a LLOYD model LS1 instrument following a test protocol and setup adapted to LIB electrodes from the standards ISO-8510, UNE-EN-28510 (Peel test for a flexible-bonded-to-rigid test specimen assembly. Part 1:90° peel) and ASTM-D3330 M and ASTM-D6862-11 for peel adhesion test methods. The maximum load of this equipment is 5 kg-f or 50 N (transductor Lloyd LC50N). The parameters of the 90° test applied are a crosshead speed of 20 mm min^–1^ and propagation speed of 20 mm min^–1^. For the instrument calibration, a mass stacking method was used, following a procedure based on standard UNE EN ISO 7500-1 (May 2018).

### Cell Assembly

The cathode disks were dried for 16 h at 120 °C under vacuum before the coin cells were assembled (10 mbar). The CR2025 cell covers (Hohsen) were cleaned with ethanol in an ultrasonic bath for 15 min and then dried for 1 h at 60 °C. The coin cells were then assembled in a dry chamber room (−40 °C dew point) utilizing the NMC 811 cathodes and graphite anodes. As electrolyte, 100 μL of 1 M lithium hexafluorophosphate in (1:1 vol %) ethylene carbonate:dimethyl carbonate + 2 wt % vinylene carbonate-99.9% (1 M LiPF_6_ in EC:DMC (1:1) + 2 wt % VC) was utilized. The separators used were glass fiber type (Whatman GF/A) with a pore size of 1.6 μm and thickness of 260 μm, which had previously been dried at 60 °C.

### Electrochemical Characterization

Galvanostatic charging and discharging cycles were conducted on the NMC 811|graphite coin cells, using a BaSyTec CTS Battery Test System, in a voltage range of 2.8–4.3 V. After 8 h rest at open circuit potential, the electrochemical response was tested at different C-rates: 0.1C, 0.5C, 1C, 2C, 3C, 5C and, finally, a cycling of 90 cycles at 0.5C. To calculate the C-rate, the theoretical capacity of NMC 811 active material was considered (200 mAh g^–1^), which was provided by the supplier. Three samples of each binder were studied.

Electrochemical impedance spectroscopy (EIS) measurements were performed in two stages, after the first cycle of formation and after the cycling with a voltage amplitude of 10 mV and a frequency range varying from 1 MHz to 1 mHz, utilizing a VMP-3 potentiostat (Biologic Science Instruments).

## Results and Discussion

**Figure 1:**
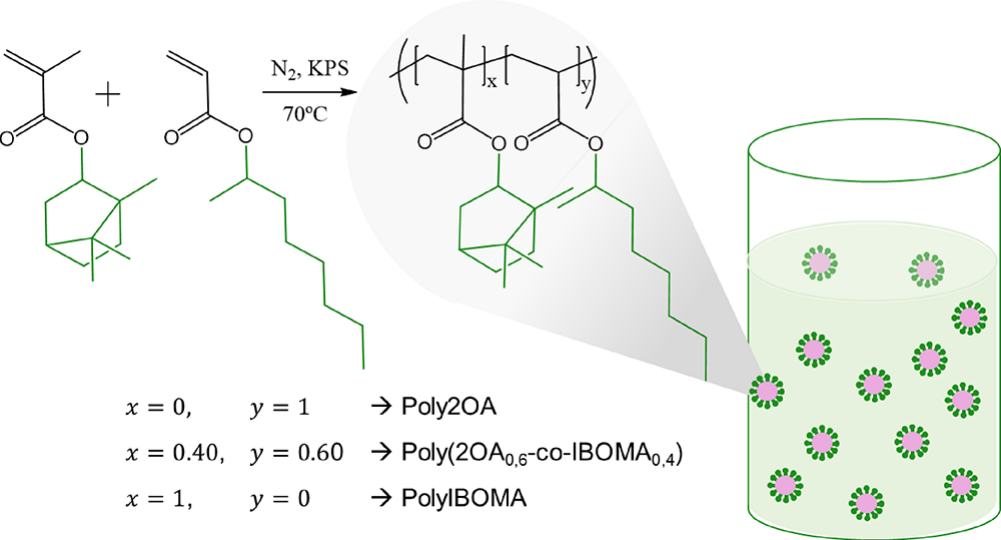
Scheme of the (co)polymerization of the (semi)batch polymerization of the biobased latexes with different compositions.

As schematized in Figure 1, three different compositions of biobased latexes were synthesized by free radical polymerization (mini)emulsion: (1) Poly2OA homopolymer, (2) Poly(2OA_0,6_-*co*-IBOMA_0,4_) random copolymer, and (3) PolyIBOMA homopolymer. In all cases, full conversion was achieved and the particle sizes were 250 nm (PDI 0.037), 230 nm (PDI 0.022), and 190 nm (PDI 0.027). The ^1^H NMR of the Poly(2OA_0,6_-*co*-IBOMA_0,4_) random copolymer is shown in Figure S1. The homopolymers of Poly2OA and PolyIBOMA present glass-transition temperatures of −44^26^ and 140–195 °C,^25^ respectively. The glass-transition temperature of the Poly(2OA_0,6_-*co*-IBOMA_0,4_) random copolymer was evaluated by a DSC test (Figure S2). The *T*_g_ of this copolymer latex resulted in 12 °C, which is slightly higher than the one calculated with the Fox eq (7.5 °C), likely due to the uncertainties on the *T*_g_ of IBOMA.

**Figure 2:**
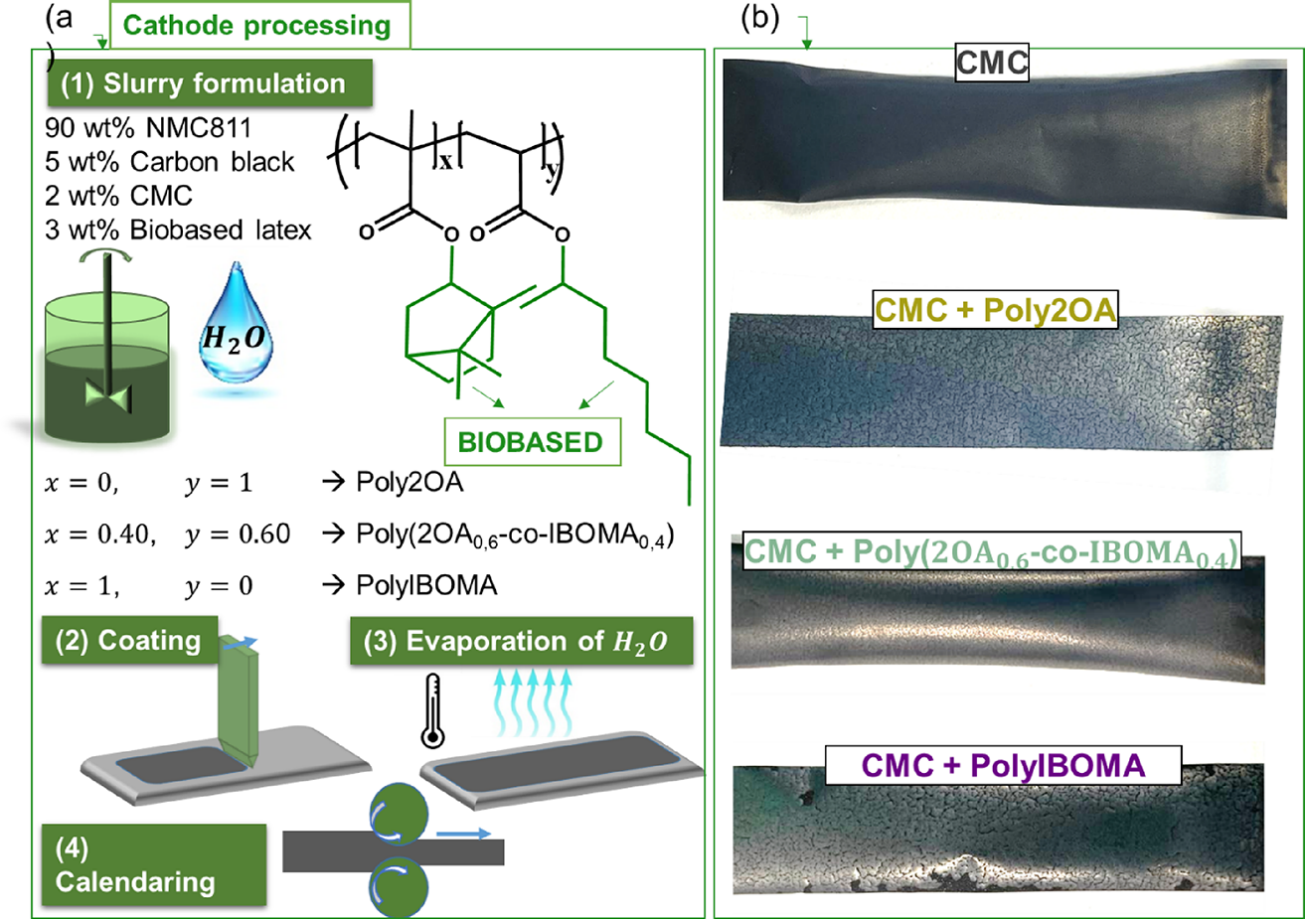
(a) Schematic representation of the processing of NMC 811 cathodes. (b) Pictures of the electrodes after calendaring using different binder compositions.

Different cathode compositions were prepared following the steps represented in Figure 2a. First, aqueous slurries were prepared using a mechanical mixer composed of 90 wt % NMC 811, 5 wt % carbon black, 2 wt % of CMC, and 3 wt % of the different biobased latexes, in addition to two more slurries with no latex, 5 wt % CMC and 5 wt % PVDF, used as a control. For the PVDF slurry preparation, NMP was employed as a solvent.

The binder plays a crucial role in dispersing the active and conductive particles and avoiding agglomerations. To assess the ability of the biobased latex to fulfill this purpose, the rheological behavior was studied. Figure S3 shows the viscosity versus shear rate curves for the slurries. Since the 5 wt % CMC slurry (no latex) has larger amount of the CMC thickener, it has the highest values of viscosity at all shear rates. As explained in the Experimental Section, one method to analyze the rheological results is to fit the curves with the power-law model, obtaining the deviation of the slurry from a Newtonian fluid, which is represented by *n* equal to 1. The more proximity to a Newtonian fluid is related to a more stable slurries.^27^ By fitting the curves in Figure S3, the *n* factor values resulted in 0.4 for CMC (no latex) and CMC + Poly2OA slurries, 0.5 PVDF and CMC + PolyIBOMA-based slurries, and, finally, 0.6 for CMC + Poly(2OA_0,6_-*co*-IBOMA_0,4_) slurry. Furthermore, the two slurries with the lower *n* factor (CMC and CMC + Poly2OA) also presented higher viscosity than CMC + Poly(2OA_0,6_-*co*-IBOMA_0,4_) and CMC + PolyIBOMA. An excessively viscous slurry is undesirable as it complicates the creation of uniform coatings and can result in material agglomeration and inhomogeneous electrodes.^28^ Hence, all slurries exhibited shear-thinning characteristics. Although the distinction is subtle, the findings suggest that the CMC + Poly(2OA_0,6_-*co*-IBOMA_0,4_) latex generates slurries with viscosity suitable for the coating process, enabling the production of more uniform and reproducible electrodes.

After casting and calendaring, the electrodes shown in Figure 2b were obtained, which have different visual aspects depending on the binder formulation. The 5 wt % CMC (no latex) electrode has a smooth surface but is fragile when handling it. On the other hand, within the electrodes containing the biobased latexes, the CMC + Poly2OA-based electrode shows the most cracked coating, which can be due to the low *T*_g_ latex used (−44 °C), which did not yield enough cohesiveness to keep all components together. When increasing the amount of IBOMA, the CMC + Poly(2OA_0,6_-*co*-IBOMA_0,4_)-based coating exhibits an enhanced homogeneity and less cracks in comparison with the other electrode strips. Nevertheless, the CMC + PolyIBOMA electrode has less cracks than CMC + Poly2OA, but fragments of the coating deattached from the current collector, probably because of the high rigidity of the coating that leads to lack of adhesiveness. Finally, the PVDF electrode presented a smooth surface without any discernible cracks or defects.

**Figure 3:**
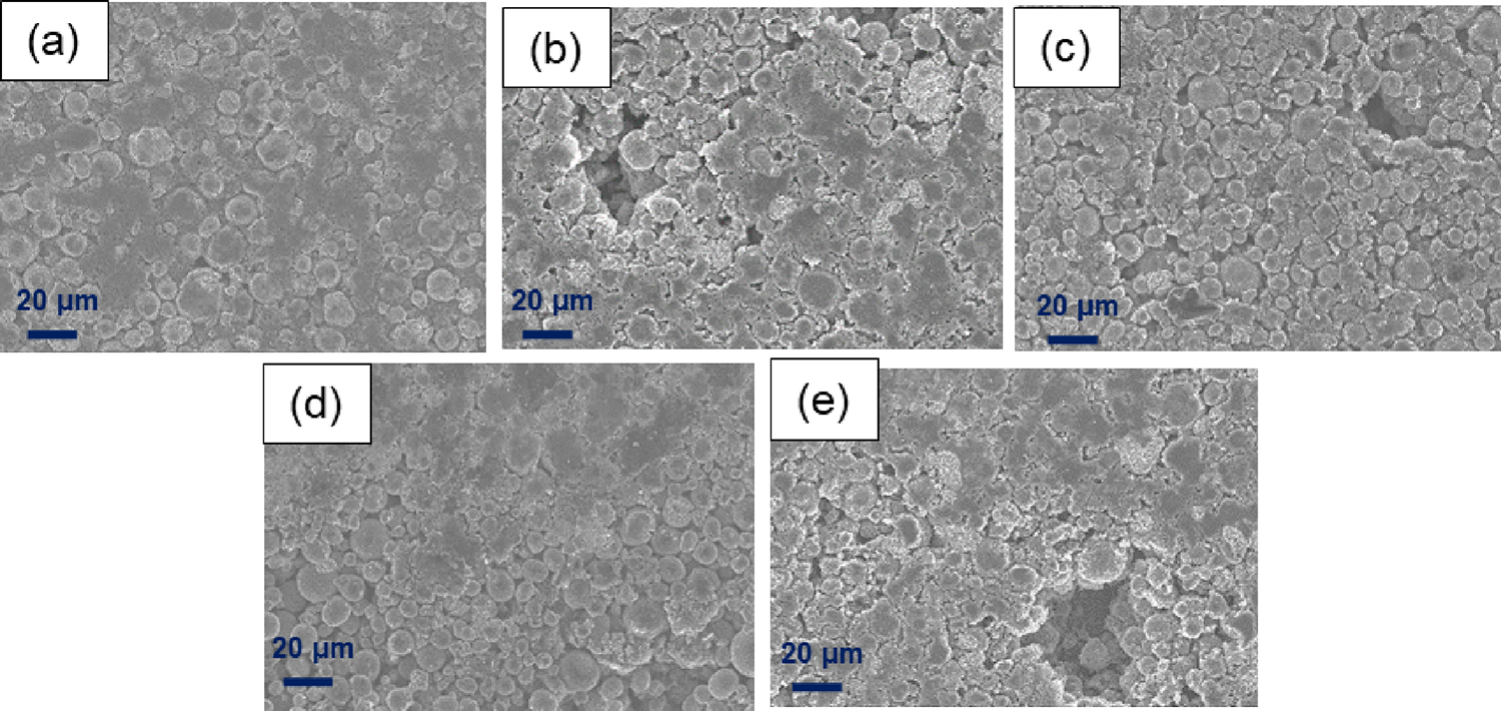
FESEM images (500×) of the surface of pristine electrodes using as binder (a) PVDF, (b) CMC with no latex, (c) CMC + Poly2OA, (d) CMC + Poly(2OA_0,6_-*co*-IBOMA_0,4_), and (e) CMC + PolyIBOMA.

Figure 3 depicts the FESEM images of the pristine electrodes (i.e., without cycling) containing different binders. The PVDF-based electrode shows a good particle distribution, and no damage is observed in the electrode surface. However, the CMC electrode with no latex addition shows voids of around 40 μm over the surface and lack of interconnection between particles that can be a consequence of the low flexibility and adhesion of the CMC binder by itself. On the other hand, the coating with CMC + Poly2OA depicted fissures along the electrode that may hinder the conductivity and that were also visually observed in the picture of the electrode strip shown in Figure 2b. Furthermore, the PolyIBOMA-based electrode presents holes even bigger than that with the CMC alone (approximately 60 μm) because of the delamination of the coating from the current collector, although particles seem to be well interconnected. Noticeably, the electrode with Poly(2OA_0,6_-*co*-IBOMA_0,4_) latex did not present any voids or holes and the coating appears to be less damaged than the other electrodes, showing good particle distribution as the PVDF electrode.

To further understand the impact of the different latexes on the cathode properties, peel tests were performed. Figure S4 shows the force (*N*) as a function of the distance curves obtained during the peeling test. The results revealed that the reference electrodes with only the CMC or PVDF as a binder presented a peeling strength of 4 ± 1 and 9 ± 1 N m^–1^, respectively. On the other hand, the latex-based electrodes showed an enhanced adhesion of 132 ± 9, 210 ± 9, and 165 ± 24 N m^–1^ for CMC + Poly2OA, CMC + Poly(2OA_0,6_-*co*-IBOMA_0,4_), and CMC + PolyIBOMA, respectively. Once more, a clear impact of the biobased latex rigidity is noticed since the peeling strength of the electrode with Poly2OA was enhanced when adding IBOMA in the latex. Notably, the peeling test of the CMC + PolyIBOMA electrode revealed a surprisingly high adhesion strength. This might be attributed to its capability to establish robust mechanical interactions between the particles and the current collector, despite its tendency for detachment due to high rigidity. This observation aligns with the comparatively fewer surface cracks evident in the CMC + PolyIBOMA electrode, as illustrated in Figure 2.

All in all, the CMC + Poly(2OA_0,6_-*co*-IBOMA_0,4_) latex demonstrated the best compromise between flexibility from the 2OA monomer and stiffness from the IBOMA, offering improved mechanical properties to achieve homogeneous electrodes with improved peeling strength. This could potentially boost the conductivity by keeping the particles closely together and creating paths for the charge–discharge process. To assess the electrochemical stability of the biobased latex within the cycling potential range, cyclic voltammetry tests were conducted by assembling cells using electrodes without active material (NMC811) and lithium metal as the anode (Figure S5). The results indicate that no degradation was observed when cycling up to 4.5 V compared to Li/Li^+^. However, subsequent cycles up to 5 V versus Li/Li^+^ revealed a noticeable increase in the oxidation peak. Hence, it is evident that latex Poly(2OA_0,6_-*co*-IBOMA_0,4_) is suitable for use as a binder within the potential range of cycling from 2.8 to 4.3 V.

**Figure 4:**
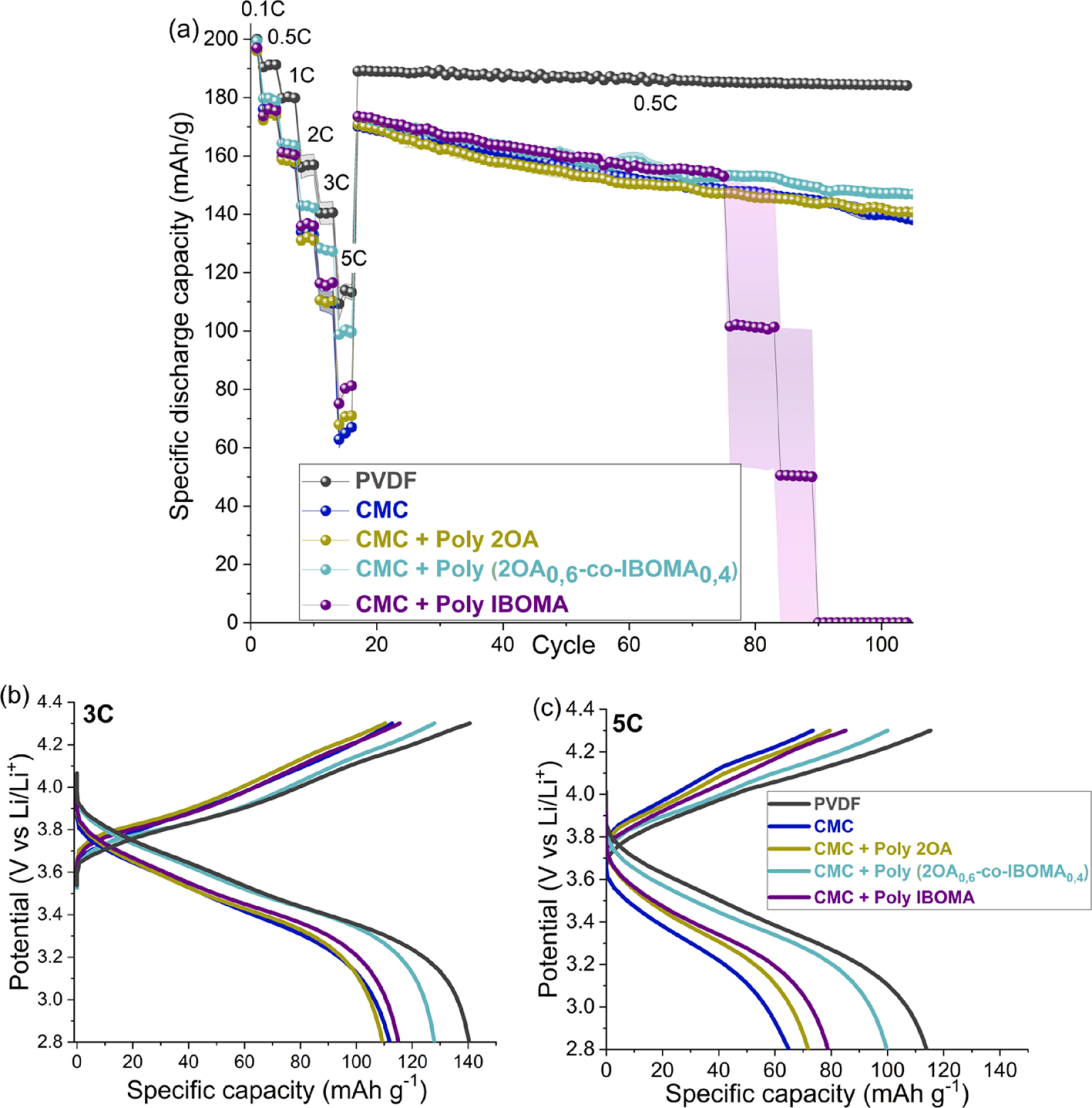
Electrochemical performance of NMC 811|graphite coin cells using different binders. (a) Rate capability performance with three cycles at 0.5C, 1C, 2C, 3C, and 5C and cycling performance at 0.5C. Voltage profiles at (b) 3C and (c) 5C. Potential range: 2.8–4.3 V at 25 °C.

Using the NMC 811 cathodes with different binders, coin cells were assembled using graphite anodes and 1 M LiPF_6_ in EC:DMC (1:1) + 2 wt % VC as a electrolyte. In the case of the PolyIBOMA-based coating, only circular electrodes with no visual delamination were selected during the coin cell assembly, trying not to damage the electrodes since they were quite fragile. Three coin cells of each electrode were assembled and the average galvanostatic cycling capacity results are shown in Figure 4 with the most relevant data summarized in Table 1. The first cycle was performed at 0.1C rate to allow the formation of a robust and stable solid electrolyte interface (SEI) on the graphite anodes.^29^ All cells delivered almost the same initial discharge capacity (between 197 and 199 mAh g^–1^) with a Coulombic efficiency around 87–88% (Figure S6). However, when the C-rate of the charge–discharge process was increased (especially at 3C and 5C) (Figure 4a), the coin cell using the CMC + Poly(2OA_0,6_-*co*-IBOMA_0,4_)-based cathode revealed an appreciably improved performance in comparison with the other aqueous cells. The cells using PVDF as a binder presented the highest discharged capacities. This may be attributed to the degradation that NMC 811 active material suffers in aqueous solutions. Therefore, this issue is avoided since no water is involved during the PVDF electrode fabrication. Instead, NMP is needed as a solvent for PVDF, which is sought to be replaced by greener solvents given its toxicity.

**Table 1:** Summarized Data of Electrochemical Performance of NMC 811|Graphite Coin Cells Using Different Cathode Binders

cathode binders	DC^a^ [mAh g^–1^] 3C cycle 12	DC^a^ [mAh g^–1^] (5C) cycle 15	DC^a^ [mAh g^–1^] (0.5C) cycle 17	CR^b^ 0.5C 90 cycles
PVDF	140.4 ± 3.8	113.9 ± 2.6	189.1 ± 0.5	96%
CMC	109.9 ± 3.4	64.5 ± 0.8	170.1 ± 1.6	81%
CMC + Poly2OA	109.7 ± 0.3	70.7 ± 1.1	170.7 ± 1.6	81%
CMC + Poly(2OA_0,6_-*co*-IBOMA_0,4_)	128.3 ± 0.1	100.3 ± 2.1	173.4 ± 1.2	84%
CMC + PolyIBOMA	115.5 ± 2.2	80.4 ± 1.2	173.5 ± 0.8	not stable

a DC = specific discharge capacity (mAh g^–1^ NMC 811).

b CR = capacity retention 90 cycles (%) = [DC_Cycle 107_] × [DC_Cycle 17_] – 1 × 100.

The overpotentials of the voltage profiles at 3C and 5C (Figure 4b,c, respectively) for the CMC + Poly(2OA_0,6_-*co*-IBOMA_0,4_) biobased latex cells were similar to those for the PVDF ones, although water was employed as a solvent for the electrode preparation. The CMC + Poly(2OA_0,6_-*co*-IBOMA_0,4_) biobased latex delivered 128.3 ± 0.1 and 100.3 ± 2.1 mAh g^–1^ at 3C and 5C while PVDF showed 140.4 ± 3.8 and 113.9 ± 2.6 under the same conditions. Surprisingly, the delivered discharge capacity of CMC + Poly(2OA_0,6_-*co*-IBOMA_0,4_) was followed by the CMC + PolyIBOMA-based cathode, although the coating detached from the current collector. Regarding the electrode using the CMC + Poly2OA biobased latex, it showed a similar performance to the CMC electrode with no latex. Following the galvanostatic cycling procedure described in the Experimental Section, after the rate capability tests, a cycling test at 0.5C is carried out. At the beginning, the discharge capacities of the different aqueous cathodes exhibit minor differences, almost 174 mAh g^–1^ for Poly(2OA_0,6_-*co*-IBOMA_0,4_) and PolyIBOMA and between 170 and 171 mAh g^–1^ for the other cathodes and around 190 mAh g^–1^ for PVDF electrodes. However, the CMC and CMC + Poly2OA electrodes suffered from a faster decay in the cycling performance with a capacity retention of 81% after 90 cycles at 0.5C. Under the same conditions, the cathodes using the CMC + Poly(2OA_0,6_-*co*-IBOMA_0,4_) binder attained a capacity retention of 84%, which was lower than for PVDF cells (96%). It is worth mentioning that although the PolyIBOMA-based coin cells showed a promising performance during the rate capability and the beginning of the cycle-life tests, the discharge capacity dropped to zero at certain points during the cycling, starting to fail from cycle 75. This behavior may be due to mechanical detachment of the coating from the current collector that hinders the electric contact required for the charge–discharge process. As mentioned previously, the combination of the soft monomer (2OA) and hard monomer (IBOMA) provides the Poly(2OA_0,6_-*co*-IBOMA_0,4_) copolymer latex the optimal properties of flexibility, cohesiveness, and adhesion that lead to well-dispersed slurries and electrodes without cracks, which in turn boost the conductivity and lithium transport during the galvanostatic cycling.

**Figure 5:**
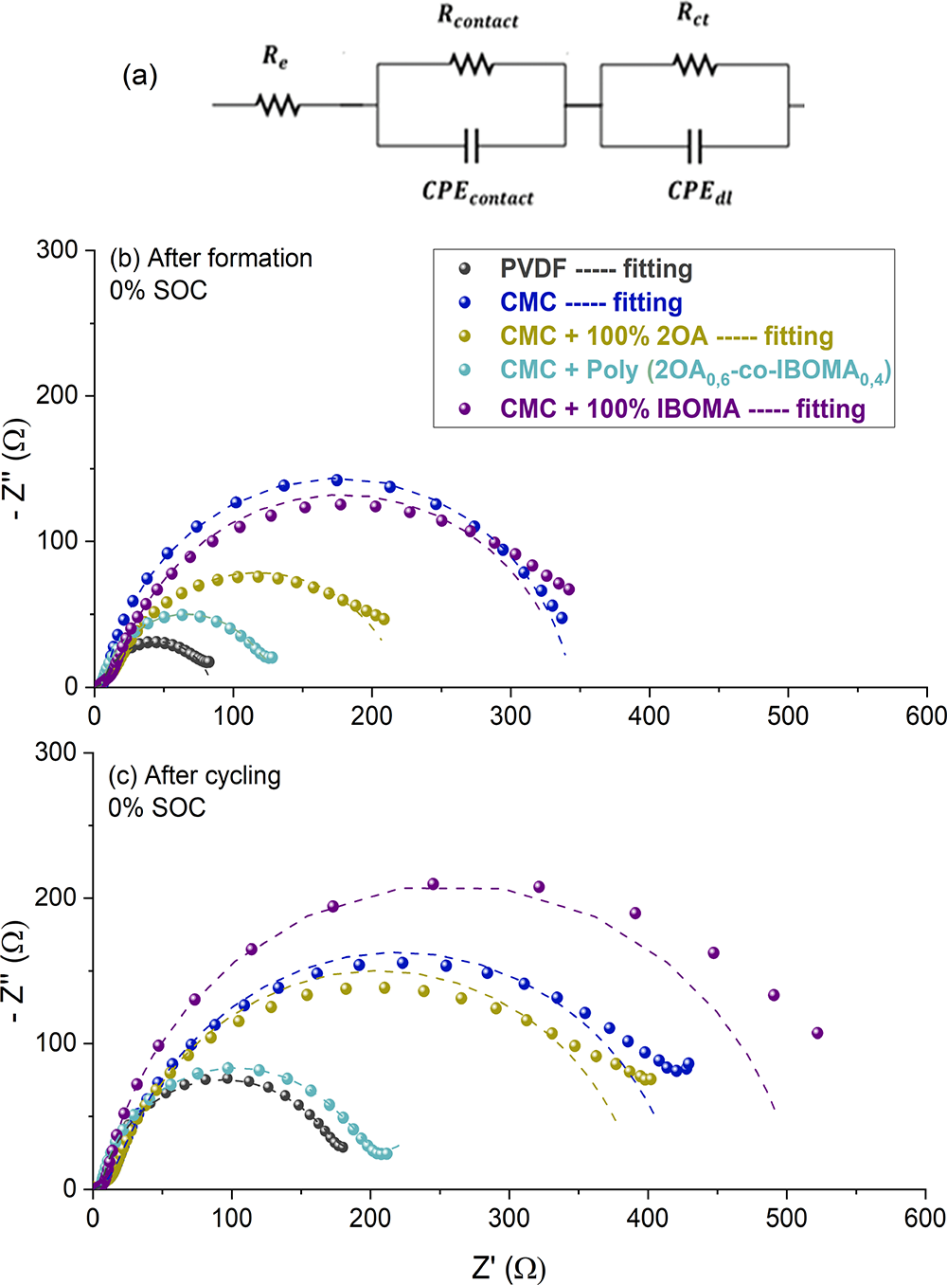
(a) Equivalent circuit for EIS fitting. Nyquist plot for (b) after formation and (c) after cycling NMC 811 coin cells.

To further understand the electrochemical performance of the different cells, EIS measurements were performed from 1 MHz to 10 mHz. Moreover, to study how these processes evolve along the cycling, the EIS tests were carried out on the discharged cells after the first formation cycle and at the end of the cycling test (C-rates + 90 cycles at 0.5C), resulting in the Nyquist plots depicted in Figure 5a,b, respectively. The equivalent circuit used to fit the EIS results is presented on the top of Figure 5 and is composed of an electrolyte resistance (*R*_e_), a contact resistance (*R*_contact_) between the current collector and the electrodes, and a charge transfer resistance (*R*_ct_) for the intercalation–deintercalation of lithium ions in the electrodes.^30^ The two latter processes are represented by double layer capacitance (CPE_constant_ and CPE_ct_, respectively). The most relevant data of the fitted Nyquist plots are presented in Table S2.

The electrolyte resistance (*R*_e_) in both the pristine and aged condition was alike for all electrodes (2Ω), indicating a small contribution of *R*_e_ to the resistance processes inside the cells. The *R*_contact_ process exhibits low values for the CMC + Poly(2OA_0,6_-*co*-IBOMA_0,4_) and PVDF electrodes with small changes between the after formation and after cycling stages (3–6 Ω). However, the other latexes showed around 15 Ω for the *R*_contact_ after formation that almost doubled after cycling, which may be a consequence of the cracks and holes observed in the FESEM images. This difference is negligible when the charge-transfer resistance process (*R*_ct_). An increase in the *R*_ct_ can be observed between pristine and aged electrodes in all cases, probably due to a degradation over cycling causing the loss in capacity retention observed. The lowest values were observed for the PVDF cells (80 ± 3 and 170 ± 9 for the after formation and after cycling, respectively) since the active material is not exposed to water, which causes particle degradation and deposition of resistance products. Nevertheless, in the case of the CMC + Poly(2OA_0,6_-*co*-IBOMA_0,4_) cell, similar values of *R*_ct_ were observed, increasing from 122 ± 3 to 185 ± 10 Ω. This behavior could be a consequence of a better stabilization of the active and conductive materials by the copolymer latex, which led to better particle interconnection and can explain the improved performance as well, observed in the galvanostatic cycling. On the other hand, as observed in Figure 5, the other aqueous cells manifested higher resistances (represented by the diameter of the semicircle), especially the CMC + PolyIBOMA with roughly 500 Ω after cycling. This is in accordance with the larger overpotentials and, therefore, worsens the performance observed during cycling. The presence of holes seen in the FESEM images for CMC, CMC + Poly2OA, and CMC + PolyIBOMA may also explain the increased resistance by hindering the contact between particles and consequently the lithium transport. In the same way, the fact that no voids developed in the PVDF and CMC + Poly(2OA_0,6_-*co*-IBOMA_0,4_) electrodes agrees with their lower resistance and overpotential values leading to enhanced electrochemical performances.

Finally, FESEM measurements were performed on the surfaces of aged electrodes (Figure S7), where no major differences in comparison with the pristine electrodes of Figure 3 were noticed. In the case of CMC + PolyIBOMA, after opening the coin cells, the coating was deattached from the current collector, and parts of it remained in the separator. The FESEM image was taken from an electrode part that remained unbroken. Large cavities were observed for the CMC and the CMC + PolyIBOMA electrodes. In agreement with the electrochemical performance and electrode characterization, the CMC + Poly(2OA_0,6_-*co*-IBOMA_0,4_) electrode showed a less damaged microstructure with no visible holes and improved contact between particles, which may explain the high electronic conductivity and the lower *R*_ct_ values that, in turn, accomplish an enhanced electrochemical performance.

## Conclusions

This work shows the potential of biobased latexes in combination with CMC as binders for water processing of NMC 811 cathodes. Three different biobased latex compositions were studied: (1) Poly2OA homopolymer, (2) Poly(2OA_0,6_-*co*-IBOMA_0,4_), and (3) PolyIBOMA. The proportion of hard (IBOMA) and soft (2OA) monomer in the composition had a clear impact on the mechanical integrity of the electrodes. The PolyIBOMA coating detached from the current collector because of its lack of flexibility and adhesion, while the Poly2OA-based electrode presented abundant cracks because it lacked cohesion. Consequently, both homopolymer-based cells delivered poor electrochemical performance. However, the combination of both proffered the optimal characteristics to the Poly(2OA_0,6_-*co*-IBOMA_0,4_) biobased latex, producing crack-free electrodes with high peeling strength, ensuring good electrode integrity, and preventing detachment of active material during cycling. Therefore, the outcome was high specific capacities at high C rates (128.3 ± 0.1 and 100.3 ± 2.1 mAh g^–1^ at 3C and 5C) and acceptable retention capacity of 84% after 90 cycles at 0.5C. The electrodes with organic PVDF and the aqueous processed Poly(2OA_0,6_-*co*-IBOMA_0,4_)-based electrodes showed the lowest polarization in the voltage profiles in comparison with the other aqueous cells. This behavior was also observed in the EIS tests by low values of the charge-transfer resistance. In summary, the findings of this research demonstrate the potential of Poly(2OA_0,6_-*co*-IBOMA_0,4_) biobased latex as a binder for NMC 811 electrodes, providing the optimal characteristics of flexibility, cohesion, and adhesion, while relying on renewable resources. Further research and development efforts in these sustainable polymers will contribute to the advancement of green-energy storage technologies.

## Supporting Information Available

^1^H NMR of the copolymer; GPC results of biobased latexes; DSC test of the random copolymer latex; rheology curves of the slurries with different binders; force vs distance curves of the peeling tests using different binders; Coulombic efficiency of the full test galvanostatic cells; fitted resistance data of the EIS Nyquist plots (PDF)
