# Supplementary material for: Biobased Acrylic Latexes/Sodium Carboxymethyl Cellulose Aqueous Binders for Lithium-Ion NMC 811 Cathodes
Source: ACS Appl Polym Mater. 2024 Jan 8;6(2):1236–44. doi: 10.1021/acsapm.3c02167 (PMC10825816; doi:10.1021/acsapm.3c02167)
Supplement: Supplementary file 1 — ap3c02167_si_001.pdf [file ap3c02167_si_001.pdf]

Supporting information

Biobased Acrylic Latexes/Sodium Carboxymethyl Cellulose Aqueous Binders for Lithium-ion NMC811 Cathodes

Ana Clara Rolandi [a,b,c], Aitor Barquero[c], Cristina Pozo-Gonzalo[a], Iratxe de Meatza[b],

Nerea Casado[c,e], Maria Forsyth[a,c,e], Jose R. Leiza[c] and David Mecerreyes[c,e]**

[a]Institute for Frontier Materials, Deakin University, Melbourne, Victoria 3125

[b]CIDETEC Basque Research and Technology Alliance (BRTA), Paseo Miramon 196, 20014 Donostia-San Sebastian, Spain

[c]POLYMAT and Applied Chemistry Department, Faculty of Chemistry, University of the Basque Country UPV/EHU, Joxe Mari Korta center, 20018 Donostia-San Sebastián, Spain.

[d]IKERBASQUE, Basque Foundation for Science, 48009 Bilbao, Spain

*To whom correspondence should be addressed.

Email: david.mecerreyes@ehu.es

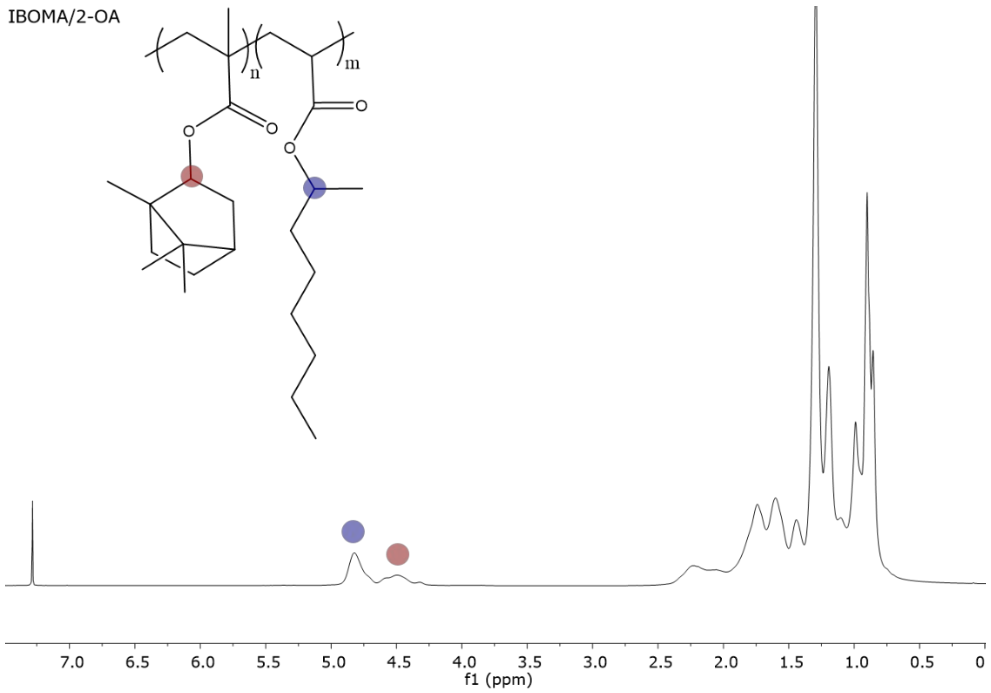

Figure S1. 1H-NMR of the Poly($2OA_{0,6}$-co-$IBOMA_{0,4}$) copolymer.

Table S1. Gel content, weight average molar mass and dispersity of the biobased polymers.

**Polymer**	**Gel content (%)**	**M_w (kg/mol)**	**Đ**
Poly2OA	75	217	2.6
Poly(2OA0,6-co-IBOMA0,4)	42	519	5.1
PolyIBOMA	0	759	3.6

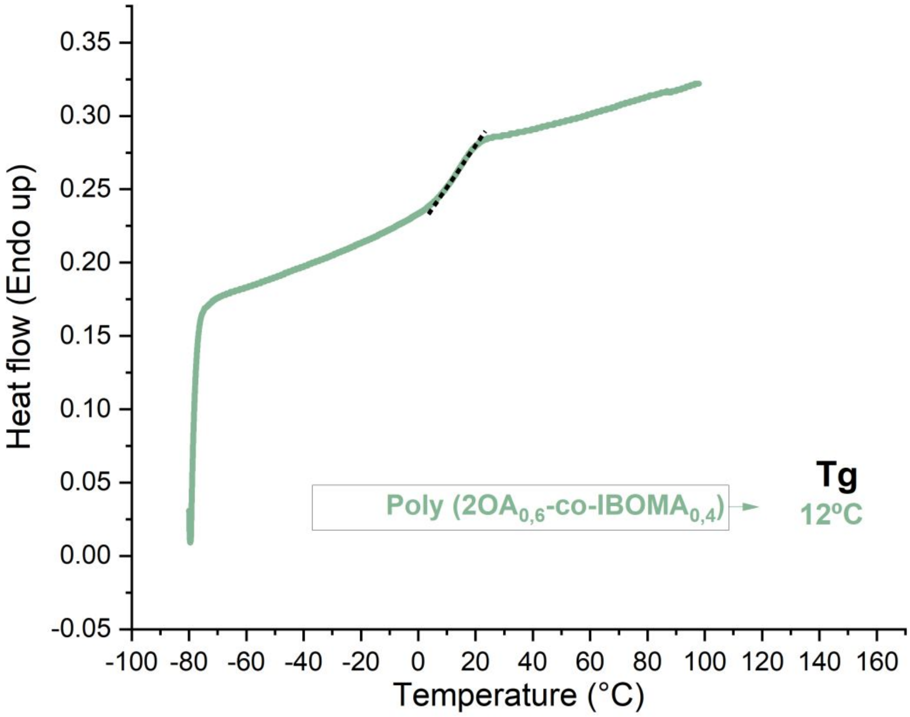

Figure S2. Differential scanning calorimetry (DSC) of the copolymer biobased latex at 10 ˚C min^{-1} in the temperature range of -80°C to 100˚C.

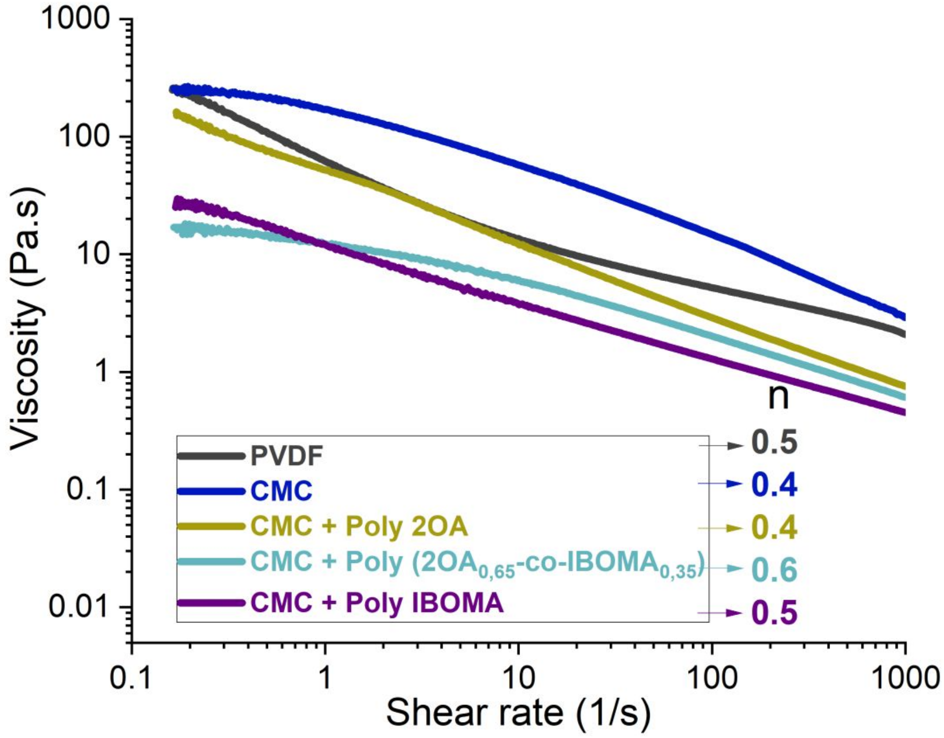

Figure S3. (a) Rheology curves of cathode slurries performed at 25˚C between 0.1 and 1000 s^{-1} shear rate.

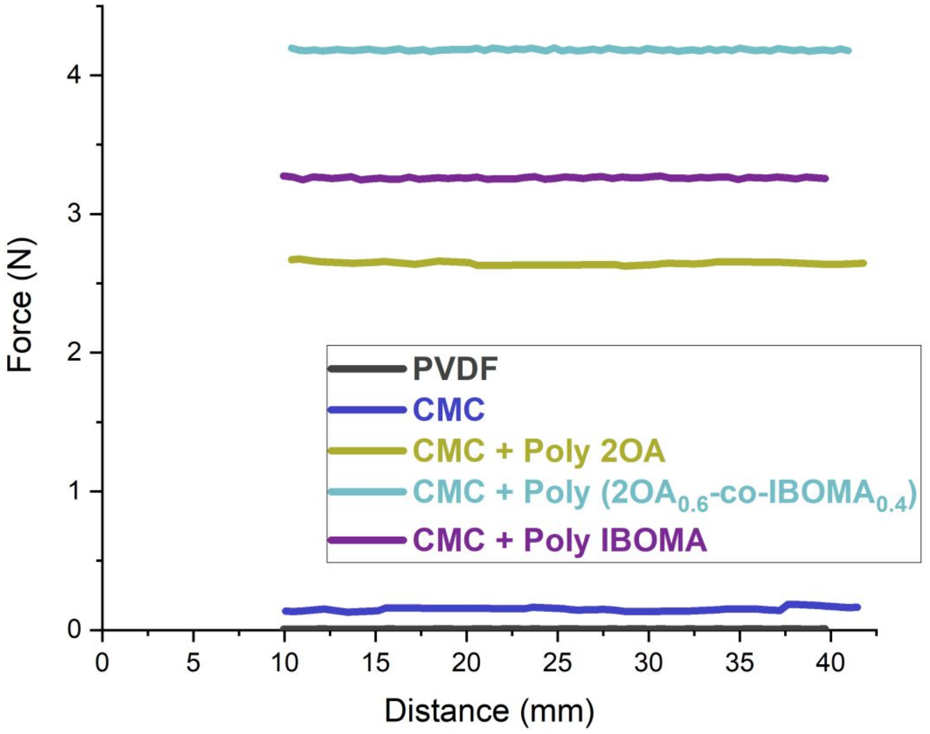

Figure S4. Peel tests of electrodes coated from the different binder slurries.

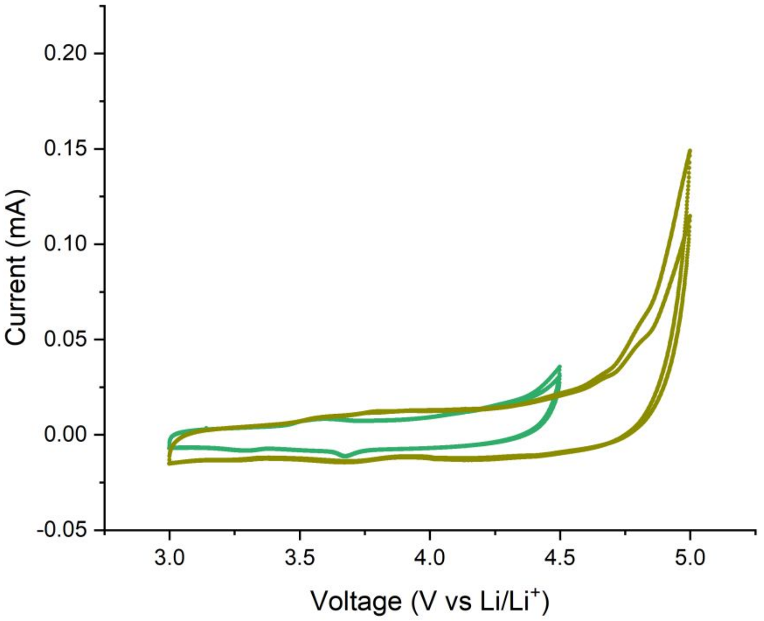

Figure S5. Cyclic voltammetry of coin cells of electrodes 50 wt% of copolymer biobased latex, 25 wt% CMC and 25 wt% conductive carbon and lithium metal as anode. 0.1 mV s^{-1} between 2.0 - 5.0 V vs Li/Li^{+}.

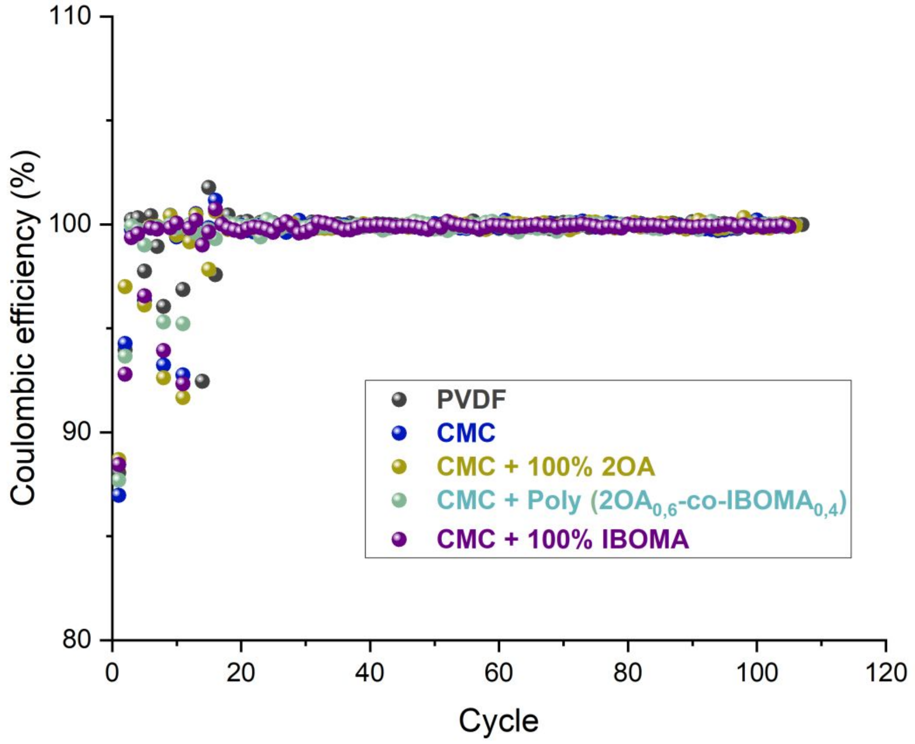

Figure S6. Coulombic efficiency of the full cells galvanostatic tests.

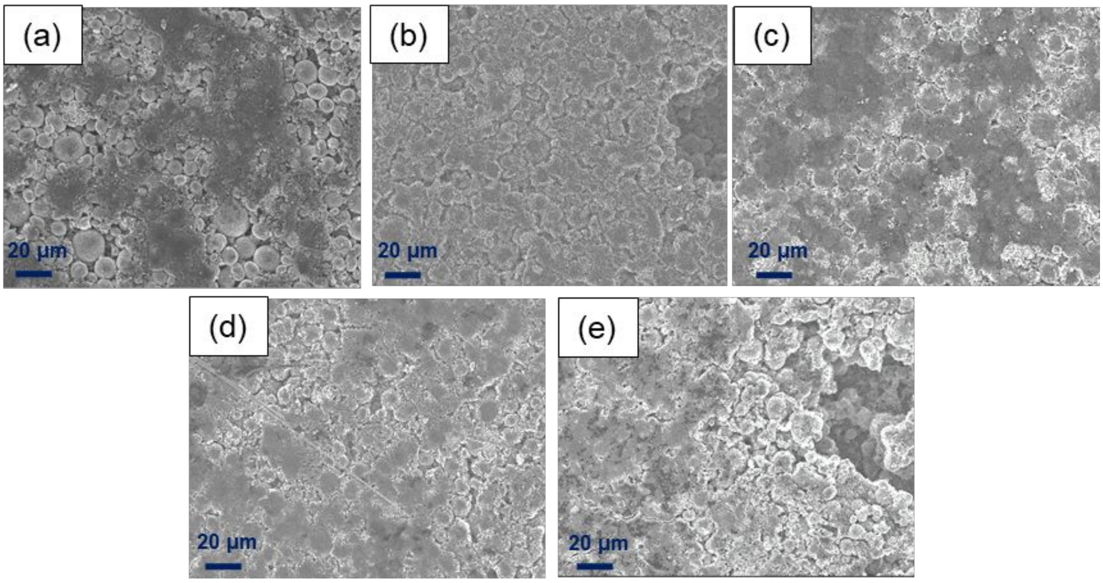

Figure S7. FESEM images (500X) of the surface of aged electrodes using as binder (a) PVDF, (b) CMC with no latex, (c) CMC + Poly2OA, (d) CMC + Poly(2OA$_{0,6}$-co-IBOMA$_{0,4}$) and (e) CMC + PolyIBOMA

Table S2. Fitted resistance data of EIS Nyquist plots.

		R_e (Ω)	$R_{contact}$ (Ω)	R_{ct} (Ω)
PVDF	Pristine	2 ± 1	4 ± 1	80 ± 3
	Aged	2 ± 1	4 ± 1	170 ± 9
CMC	Pristine	2 ± 1	16 ± 1	337 ± 7
	Aged	2 ± 1	29 ± 8	396 ± 18
CMC + Poly2OA	Pristine	2 ± 1	16 ± 5	205 ± 9
	Aged	2 ± 1	23 ± 7	371 ± 16
CMC + Poly(2OA0,6-co-IBOMA0,4)	Pristine	2 ± 1	3 ± 1	122 ± 3
	Aged	2 ± 1	5 ± 1	185 ± 10
CMC + PolyIBOMA	Pristine	2 ± 1	15 ± 3	340 ± 14
	Aged	2 ± 1	30 ± 6	497 ± 30
